# Magnetic resonance imaging of the fetal brain at 3 Tesla: Preliminary experience from a single series

**DOI:** 10.1097/MD.0000000000012602

**Published:** 2018-10-05

**Authors:** Nivaldo Adolfo da Silva, José Vassallo, Luis Otávio Sarian, Christophe Cognard, Annick Sevely

**Affiliations:** a Department of Radiology, Faculty of Medical Sciences, State University of Campinas, Campinas (UNICAMP), Campinas-SP, Brazil; b Department of Diagnostic and Therapeutic Neuroradiology, Centre Hospitalier Universitaire (CHU) - Hôpital Pierre Paul Riquet, University of Toulouse, Toulouse, France; c Department of Neuroradiology, Diagnósticos da América (DASA Group), São Paulo; d Laboratory of Molecular and Investigative Pathology-LAPE, Faculty of Medical Sciences, State University of Campinas (UNICAMP), Campinas-SP, Brazil; e Department of Obstetrics and Gynecology, Faculty of Medical Sciences, State University of Campinas (UNICAMP), Campinas-SP, Brazil.

**Keywords:** 3 T, cerebral malformations, fetal brain, high-field magnetic resonance imaging, magnetic resonance imaging

## Abstract

To report our preliminary experience with cerebral fetal magnetic resonance imaging (MRI) with a 3 Tesla (3T) scanner. We assessed feasibility, time of acquisition, and possibility to establish a diagnosis.

Fifty-nine pregnant women had fetal MRI performed during the third trimester of pregnancy due to clinical or sonography concern of a central nervous system anomaly. No fetal or maternal sedation was used. The MRI protocol consisted of T2 turbo-spin-echo images in 3 planes of space. No T1-weighted images were performed. All images were analyzed by 2 pediatric neuroradiologists, who evaluated spatial resolution, artifacts, time of acquisition, and possibility to establish a diagnosis suspected by sonography.

Examinations were performed safely for all patients. The images required longer time of acquisition (approximately 75 seconds for each plane in the space). The specific absorption rate was not exceeded in any fetus. Cerebral fetal MRI was normal in 22 cases. The spectrum of diagnostics included isolated ventriculomegaly, posterior fossa malformation, corpus callosum malformation, gyration anomalies, craniosynostosis, tuberous sclerosis, microcephaly, external hydrocephaly, midline arachnoid cyst, cerebral lesions, and persistent hyperplastic primitive vitreous.

In our series, 3 T MRI of fetal brain was feasible and able to establish a diagnosis but required longer time of acquisition.

## 1 Introduction

Magnetic resonance imaging (MRI) is a powerful complementary diagnostic tool to prenatal ultrasonography (US) in elucidating morphological fetal anomalies. The higher spacial resolution aids decision making during pregnancy and management of the delivery.

Since its introduction in prenatal diagnosis in 1983,^[1]^ the technique of fetal MRI has evolved from low strength MRI scanners (0.6 T) to the development of ultrafast sequences as HASTE (half Fourier single-shot turbo spin echo), which allowed the evaluation of the highly mobile fetus. Currently, fetal MRI is often performed in 1.5 T MRI scanners. All these achievements were in accordance with the recommendations of the National Radiological Protection Board,^[2]^ International Non-Ionizing Radiation Committee of the International Radiation Protection Association (International Commission on Non-Ionizing Radiation Protection),^[3]^ and in a white paper on MRI safety.^[4]^

In 2002, the use of 3 T magnets for human imaging was approved by the United States Food and Drug Administration.^[5]^ Theoretically, MR scanners operating at 3T provide higher spatial resolution, and better quality images with faster acquisitions, thereby allowing many clinical applications in neuroradiology, body imaging, and assessment of the musculoskeletal system.^[6]^

It is challenging to obtain MRI images due to the fetuses’ small dimensions and virtually ceaseless movements. In lower-strength MRI scanners, with longer times of acquisition and lower spatial resolution, these characteristics inherent to most fetuses lead to poor quality images. In fetal medicine, higher magnetic field scanners (1.5 T MRI scanners or above), with higher signal to noise ratio have several advantages over scanners with weaker magnetic fields. These include the possibility of obtaining sharp images from small anatomic structures, to decrease time of acquisition, and avoid motion blur.

Some articles describe 3 T MRI for body fetal imaging, but data regarding the caveats of imaging the fetal brain are scarce^[7,8]^ 3T MRI has promising results in terms of better depiction of small anatomic structures (like inner ear and posterior fossa), although some disadvantages as increased magnetic field heterogeneity and standing wave artifacts have been described.^[7,8]^ The scarce literature on the specific application of 3 T MRI in the study of the fetal brain, in which encouraging results in terms of quality of imaging are balanced against inconveniences such as artifacts, prompted us to report our preliminary experience in cerebral fetal 3 T MRI. We used a 3-tiered approach to describe our experience: feasibility, time of acquisition, and possibility to establish a diagnosis.

## 2 Materials and methods

From September 2008 to March 2009, 59 patients were referred to the Department of Neuroradiology of the Centre Hospitalier Universitaire of the University of Toulouse (France) to undergo cerebral fetal MRI due to clinical or sonographic findings placing the fetus at risk for a central nervous system anomaly. US was performed by obstetricians at our referral center (Multidisciplinary Center of Prenatal Diagnosis of the University of Toulouse, France). Indications for MRI included ventricular asymmetry, posterior fossa anomalies, suspicion for cerebral malformation, previous abnormal pregnancy, and infectious antecedents like cytomegalovirus (CMV) or toxoplasmosis.

When a brain morphological anomaly was diagnosed or suspected at fetal US, the indication of each fetal MRI were discussed with a multidisciplinary team, composed by a group of physicians from several specialties, including Obstetrics, Genetics, Pediatrics, Surgery, Radiology, Pathology, and Psychology, who were involved in the management of high-risk pregnancy. Moreover, the patients were completely informed about benefits and theoretical risks of the examination, and their written consent was necessary before fetal brain MRI.

In the period between September 2008 and March 2009, all examinations were performed with a 3 T scanner (Achieva; Philips Medical Systems, Best, The Netherlands). Before that period, all examinations were done in a 1.5 T scanner which was replaced by a 3 T scanner. This latter then became the only magnet available to perform all examinations at our department.

The examinations required a 16-channel phased-array coil. The fetal brain was imaged in 3 orthogonal planes of space (roughly coronal, axial, and sagittal with respect to the fetal head). Each plane was imaged by T2-weighted turbo-spin echo single-shot images (time of repetition 15,000 ms, time of echo 141 ms, number of slices = 13 or 15, slice thickness = 5 or 4 mm, reconstruction matrix =400, acquisition matrix = 388 × 248, reconstruction voxel = 0.9, gap = 0, number of acquisitions = 1, time of acquisition = 75 seconds. No T1-weighted images were performed. No fetal or maternal sedation was used. No oxygen was delivered to the mother. In cases of discomfort, the patient was put on her side especially when polyhydramnios was present. Patients were asked to immediately signal if they sensed any heat, particularly in the abdomen. In cases of claustrophobia the examination was not performed. If motion artifacts were observed during examination, the sequences were repeated until diagnostic images were obtained.

All images were reviewed by 2 pediatric neuroradiologists (NAdS and AS) for: gyration and sulcation, encephalic biometry, brain MRI signal, and morphology. The landmarks of the cerebral development were compared to an anatomical atlas of the developmental brain^[9]^ and a fetal MRI atlas.^[10]^

Some newborns had imaging follow-up by US or MR. If the medical interruption of pregnancy was decided, some fetuses were autopsied after parental consent.

## 3 Results

**Figure 1:**
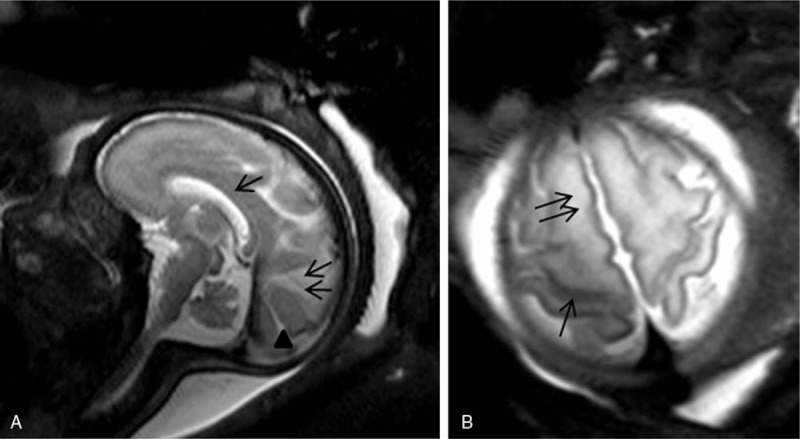
Magnetic resonance imaging (MRI) of the fetal brain at 32 weeks gestation. Sagittal T2 turbo-spin echo (TSE) single-shot image shows the corpus callosum (arrow), the parieto-occipital (double arrows) and calcarine (arrowhead) sulcus [A, Axial T2 TSE single shot image discloses the central sulcus (arrow); B, the superior frontal gyrus (double arrows)].

In 59 patients, 60 cerebral fetal MRIs were performed during a period of 6 months. The lowest gestational age was 27 weeks, and the highest was 36 weeks. MRI scans were able to depict the landmarks of sulcation and gyration according to fetal age, the signal intensity of the cerebral parenchyma and cephalic biometry. It was possible to visualize the Sylvian fissure, central sulcus, the interhemispheric fissure, parieto-occipital, and calcarine sulcus (Fig. 1A, B).

**Figure 2:**
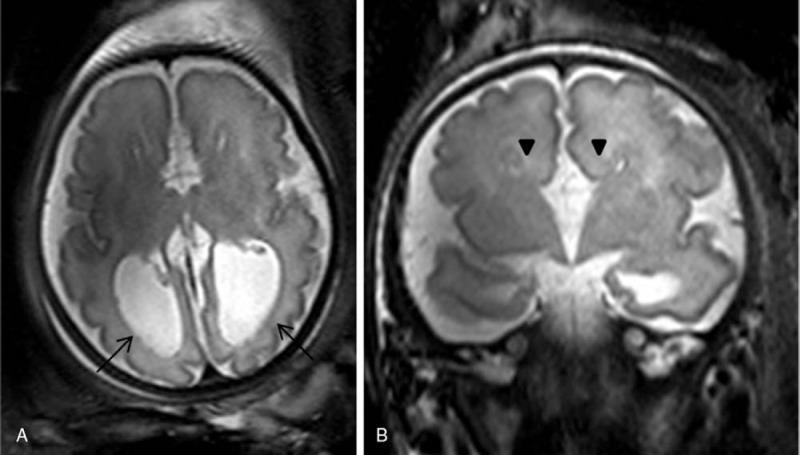
Agenesis of the corpus callosum in a fetus at 35 weeks gestation. Axial and coronal T2 turbo-spin echo (TSE) single-shot magnetic resonance imaging (MRI) scans show absence of the corpus callosum and colpocephaly (arrows, A). Probst bands (arrowheads, B).

**Figure 3:**
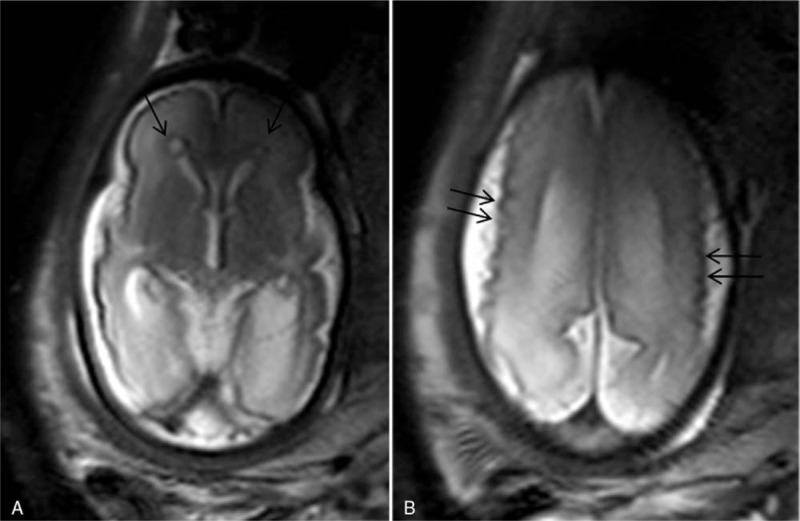
A 33 weeks gestation patient was referred for magnetic resonance imaging (MRI) because a positive cytomegalovirus serology at the first pregnancy trimester, and cerebral fetal lesions viewed by sonography (not shown). Axial T2 turbo-spin echo (TSE) single-shot images shows microcephaly, micropolygyria (double arrows), and periventricular cavities (arrows) (A and B).

**Figure 4:**
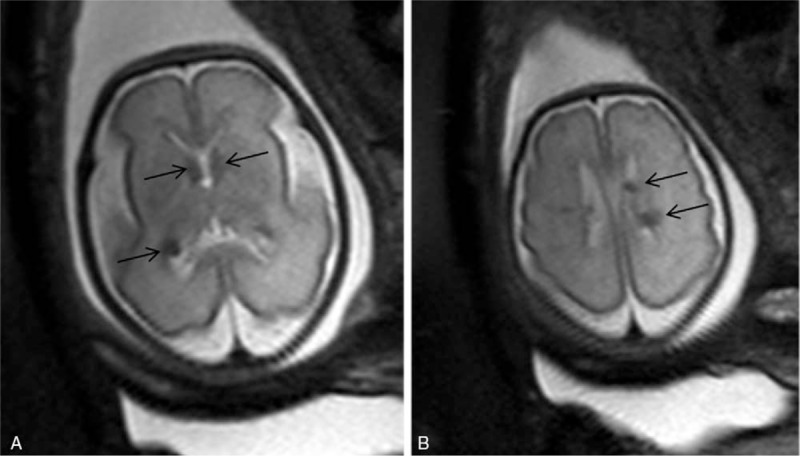
Cardiac rhabdomyomas were diagnosed by sonography in a 28 weeks gestation suggesting tuberous sclerosis but the fetal brain sonogram was normal. Axial T2 turbo-spin echo (TSE) single-shot magnetic resonance (MR) images show round hypointense (arrows) images located at the ependyma and adjacent to the Monro foramen, confirming Tuberous Sclerosis with cerebral lesions (A and B). These findings were confirmed at autopsy.

Brain fetal 3 T MRI showed no abnormalities in 22 cases. The most frequent abnormalities found in the abnormal cases were isolated ventriculomegaly (12 cases), posterior fossa anomalies including the cisterna magna and arachnoid cyst (8 cases), 6 cases of agenesis of the corpus callosum (Fig. 2A, B). Other less frequent anomalies included microcephaly and gyration anomalies (Fig. 3A, B), craniosynostosis, tuberous sclerosis (Fig. 4A, B), and persistent hyperplastic primary vitreous. These results are summarized in Table 1.

**Table 1:**
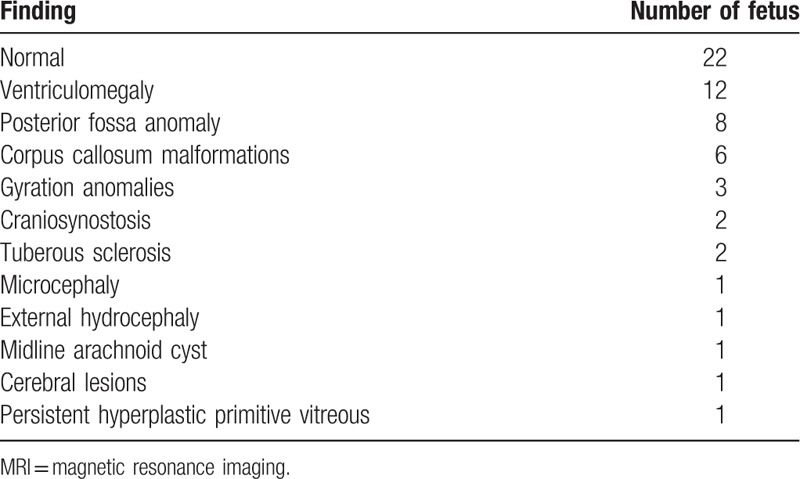
Distribution of the findings of 3 T brain fetal magnetic resonance imaging.

A total of 12 fetuses had severe morphological abnormalities that are typically associated with poor neurological outcome and warranted medical interruption of pregnancy. Ten fetuses had autopsy analysis that confirmed the principal anomaly depicted at prenatal MRI. For 2 fetuses, there were minor discordances between MR and autopsy results. In the first fetus, prenatal MR disclosed complete corpus callosum and septum pellucidum agenesis, but the postmortem study showed partial corpus callosum agenesis. In the second study, prenatal MR disclosed a large interhemispheric cyst, hydrocephalus, and hypoplasia of the cerebellar hemispheres; autopsy confirmed all findings but disclosed a rhombencephalosynapsis.

Sixteen children who had normal cerebral fetal MRI or mild anomalies (eg, minor ventriculomegaly or posterior fossa arachnoid cyst) were followed up trough postnatal ages of 16 and 22 months. All were clinically and neurologically normal.

## 4 Discussion

Since its introduction as an adjunct tool in prenatal diagnosis,^[1]^ MRI has been used for its superior contrast and spatial resolution to assess brain abnormalities suspected in prenatal sonography.^[11–14]^ In spite of the better anatomic imaging, MRI has limitations due to artifacts produced by fetus movements.^[15]^ Progressive technical developments like ultrafast sequences (fast spin-eco, HASTE, and echo-planar imaging) allowed high-quality MRI.^[16]^ 3 T MRI scanners opened new horizons and challenges especially in pediatric imaging^[17]^ provided by their theoretical high-resolution images with faster acquisitions. The increased field strength could therefore be used to obtain high-quality images of complex anatomical features of the fetus.^[18,19]^ This encouraged us to evaluate the performance of a 3 T scanner for imaging the fetal brain.

In our series, 3 T MRI was performed without complications and able to establish a diagnosis. Contrary to the expected gain in time of acquisition of the sequences at 3 T scanners, the main inconvenience that we found was the longer time of acquisition (75 seconds for each plane of the space). This was a disadvantage because it could allow artifacts due to movements of the fetus. To illustrate this, it is worth mentioning that our 1.5 T MRI scanner was able to perform 5 slices T2 HASTE sequences in 10 seconds. This difference might be explained by adjustments and specific absorption rate (SAR) management performed by the MRI software. In fact, the SAR level can be adjusted from low to high; with a lower SAR level, the system automatically increases the number of slice packages to enable more heat dissipation. This consequently increases the acquisition time.

In our study, upfront comparisons between 1.5 and 3 T imaging were no feasible since we did not perform examinations using 1.5 and 3 T scanners in the same subjects. Therefore, we are unable to ascertain whether exam accuracy is superior using images acquired using 3 T scanners. In a study^[7]^ performed to evaluate brain structures in fetuses imaged at 3 T compared to 1.5 T significantly better anatomic resolution of posterior fossa structure was obtained with the 3 T magnet. However, for the other structures assessed in the supratentorial compartment, there were no significant differences in images obtained with the 1.5 or 3 T magnets.

In our study, there was a high proportion of normal fetal brain MRI. Two reasons may help explain the high proportion of normal examinations: many patients were studied by MRI, despite a normal fetal US, because a brain malformation was found in a prior pregnancy; if there was seroconversion for CMV or toxoplasmosis during the first gestational trimester, brain fetal MRI was performed systematically, even if a recent US was normal.

Ventriculomegaly was the most frequent pathological condition diagnosed in our series (12 cases), which is compatible to what was reported in previous studies.^[20]^ Ventriculomegaly was defined if the atrial width was >10 mm at the level of the choroid glomus in an axial section that included the thalami.^[11,21]^ Fetal MRI can detect morphological anomalies that are sonographically occult in up to 40% to 50% of cases of isolated ventriculomegaly.^[20]^ In our study, some cases of ventriculomegaly (detected by prenatal sonography) 3 T MRI diagnosed associated malformations as corpus callosum dysgenesis (6 cases), arachnoid cyst (1 case), and posterior fossa anomalies (8 cases). In addition, in our study 3 T MRI was useful in diagnosing cerebral cortical anomalies, such as microcephaly, gyration anomalies and destructive lesions (3 cases and 1 case, respectively).

The major concern regarding fetal MRI is the theoretical harmful effects to the fetus due to the electromagnetic fields.^[22–24]^ It is therefore recommended to perform prenatal MRI after the first trimester of gestation to avoid the critical period of embryogenesis.^[25]^ Several experimental studies in animals examined the effects of the MRI field and gradient strengths and the radiofrequency pulses, but no definite evidence of deleterious effects was found.^[22,26]^ However, because long-term postnatal prospective studies are lacking, the international standard (IEC 60601-2-33, 2002) expresses caution for imaging pregnant women and states that there is no conclusive evidence to establish safety.^[22]^

SAR is an important safety parameter for 3 T MRI. It is a measure of the amount of energy deployed by a radiofrequency field in a determined mass of tissue. The SAR increases with field strength, so using equivalent parameters, there is a 4-fold increase in SAR at 3 T compared to 1.5 T.^[17]^ This would create problems with body temperature control, which is undesirable in the fetus due to the potential teratogenic effects of temperature elevation.^[27]^ In our study, the SAR was not exceeded in any fetus. In another study^[8]^ comparing the SAR and image quality of the MRI of the fetal brain at 1.5 and 3 T, signal-to-noise ratio was significantly higher and whole-body SAR was significantly lower for images obtained at 3 T compared to 1.5 T.

A main limitation of our work is that the temperature of the patients was not measured during MRI. This was an important safety concern. The most precise method is measure rectal temperature during examination. However, this is not a practical way and the device may cause images artifacts mainly in the pelvis. Fetus temperature measurement in MRI can be done in 2 ways: phase measurement of the signal or spectroscopy with the chemical shift measurement of N-acetyl-aspartate.^[28,29]^ These 2 techniques need a very long previous calibration, abacus establishment, and a total motionless object. This is why fetal temperature measurements during MRI are restricted to research protocols. In addition, it is needed to work with a fixed receiver gain. Therefore, we considered determining fetal temperature impractical.

We logged up to 22 months of follow-up for 16 newborns who underwent 3T cerebral MRI. All patients were neurologically normal at that point. However, we acknowledge that longer-term follow-up may be necessary to ascertain cerebral fetal 3 T MRI safety.

## 5 Conclusion

In our study 3 T MRI of fetal brain was feasible and able to establish a diagnosis but required longer time of acquisition.

## Author contributions

**Conceptualization:** Nivaldo Adolfo da Silva Jr, José Vassallo, Luis Otávio Sarian, Christophe Cognard, Annick Sevely.

**Formal analysis:** Nivaldo Adolfo da Silva Jr, José Vassallo, Luis Otávio Sarian, Christophe Cognard, Annick Sevely.

**Investigation:** Nivaldo Adolfo da Silva Jr.

**Methodology:** Nivaldo Adolfo a Silva Jr, José Vassallo, Luis Otávio Sarian, Christophe Cognard, Annick Sevely.

**Project administration:** Nivaldo Adolfo da Silva Jr.

**Supervision:** José Vassallo, Luis Otávio Sarian, Christophe Cognard, Annick Sevely.

**Validation:** José Vassallo, Luis Otávio Sarian, Christophe Cognard, Annick Sevely.

**Visualization:** José Vassallo, Luis Otávio Sarian, Christophe Cognard, Annick Sevely.

**Writing – original draft:** Nivaldo Adolfo da Silva Jr.

**Writing – review and editing:** Nivaldo Adolfo da Silva Jr, José Vassallo, Luis Otávio Sarian, Christophe Cognard, Annick Sevely.
